# Dynamics of Stress-Driven Two-Phase Elastic Beams

**DOI:** 10.3390/nano11051138

**Published:** 2021-04-28

**Authors:** Marzia Sara Vaccaro, Francesco Paolo Pinnola, Francesco Marotti de Sciarra, Raffaele Barretta

**Affiliations:** Department of Structures for Engineering and Architecture, University of Naples Federico II, Via Claudio 21, 80125 Naples, Italy; marziasara.vaccaro@unina.it (M.S.V.); francescopaolo.pinnola@unina.it (F.P.P.); marotti@unina.it (F.M.d.S.)

**Keywords:** free vibrations, nanostructures, size effects, stress-driven mixture model, integral elasticity, MEMS/NEMS

## Abstract

The dynamic behaviour of micro- and nano-beams is investigated by the nonlocal continuum mechanics, a computationally convenient approach with respect to atomistic strategies. Specifically, size effects are modelled by expressing elastic curvatures in terms of the integral mixture of stress-driven local and nonlocal phases, which leads to well-posed structural problems. Relevant nonlocal equations of the motion of slender beams are formulated and integrated by an analytical approach. The presented strategy is applied to simple case-problems of nanotechnological interest. Validation of the proposed nonlocal methodology is provided by comparing natural frequencies with the ones obtained by the classical strain gradient model of elasticity. The obtained outcomes can be useful for the design and optimisation of micro- and nano-electro-mechanical systems (M/NEMS).

## 1. Introduction

The modelling and design of advanced small-scale structures is a topic of major interest in nanoengineering. Many recent contributions in the literature concern the analysis and optimisation of new-generation composites and devices such as: biosensors [1], DNA-based sensors [2], nano/micro-resonators [3], energy harvesters [4], cantilever-based MEMS/NEMS [5], nanogenerators [6], and nanocomposites [7,8].

Analysis of micro and nanostructures has to be carried out by adequately modelling the effect of molecular interactions and inter-atomic forces which are technically significant. These long-range interactions result in size effects which cannot be overlooked. Continuum mechanics can be conveniently exploited to capture these small-scale phenomena and to predict the size-dependent responses of structural components of smaller and smaller devices, provided that some internal characteristic lengths are properly accounted for. Thus, nonlocal models of elastic continua can be conveniently adopted in place of atomistic methodologies which are computationally expensive.

Seminal works on nonlocal mechanics were contributed in [9,10,11]. Subsequently, Eringen formulated a nonlocal model of elasticity based on a strain-driven integral convolution efficiently applied to screw dislocation and wave propagation problems involving unbounded domains [12,13].

Thanks to the choice of a special averaging integral kernel, the strain-driven integral model was reversed by Eringen himself, providing a simpler, but equivalent, differential formulation. However, when applied to bounded structural domains, Eringen’s strain-driven model leads to ill-posed problems due to incompatibility between constitutive law and equilibrium requirements [14].

To bypass the aforementioned issues, a mixture strain-driven theory was developed, based on a convex combination of local and nonlocal contributions. First proposed by Eringen in [15], strain-driven two-phase theory has been recently applied in several studies, such as [16,17,18,19,20].

However, for a vanishing local fraction, Eringen’s mixture leads to singular structural problems [21]. All difficulties related to Eringen’s purely nonlocal model are overcome by the stress-driven nonlocal model [22,23,24,25] extended by the stress-driven two-phase elastostatic theory that, unlike the local/nonlocal strain-driven mixture, provides a well-posed methodology for any local fraction [26,27]. Since local/nonlocal mixtures are able to model both stiffening and softening behaviors of small-scale structures [18], they can be conveniently adopted to solve applicative problems of nanoengineering.

Thus, the motivation of the present research is to develop a well-posed stress-driven two-phase methodology to model the size-dependent dynamic behaviour of small-scale elastic beams, generalising the treatment contributed in [28] confined to stress-driven purely nonlocal nanomaterials. The proposed analytical approach, here applied to solve exemplary 1-D structural problems of technical interest, provides an advancement in nonlocal dynamics of beams with respect to the state of the art. Theoretical predictions obtained in the present research are in agreement with experimental evidence regarding small-scale inflected beams exposed in [29,30]. Extension of the stress-driven elasticity mixture to size-dependent buckling and dynamic problems of advanced materials and 2-D structures, such as graphene nanoribbons [31], will be contributed in a forthcoming paper.

The plan is the following. Kinematics and equilibrium of slender beams are preliminarily recalled in Section 2. There, the mixture stress-driven model of integral elasticity and its equivalent differential formulation are also provided. The associated differential problem governing free bending vibrations of two-phase elastic beams is formulated in Section 3. A parametric study is accomplished in Section 4 to examine the size-dependent vibrational responses of simple structural schemes of technical interest. Comparisons with outcomes obtained by the strain gradient elasticity theory are also performed and discussed in detail. Closing remarks are provided in Section 5.

## 2. Stress-Driven Mixture of Integral Elasticity

Let us consider a slender straight beam under flexure. L indicates the beam length, m denotes the mass per unit length and K is the local elastic bending stiffness, i.e., the second moment of Euler–Young moduli field E on the beam cross-section.

The bending plane is described by the Cartesian axes (x,y), with x coinciding with the beam axial abscissa. Denoting with v:[0,L]↦R the transverse displacement field of beam axis, the kinematic hypothesis of Bernoulli–Euler theory prescribes that the linearised geometric curvature field $\chi_{t}:\left[0,L\right]\mapsto R$ at a time t is related to displacements as follows

(1) $$\chi_{t}=v^{\left(2\right)}=\chi^{el}+\chi^{nel}\;,$$

where the symbol ${\left(•\right)}^{\left(n\right)}$ denotes n-times differentiation along the beam axis x. In Equation (1), $\chi^{el}$ is the elastic curvature and $\chi^{nel}$ stands for all other non-elastic curvature fields. Hereinafter, dependence on time is omitted for the sake of brevity. Equilibrated stress fields in Bernoulli–Euler beams are described by bending moments M:[0,L]↦R fulfilling the differential equation of d’Alembert dynamic equilibrium, that is:

(2) $$M^{\left(2\right)}=q-m\;\ddot{v}\;,$$

where q is a transversely distributed loading and a superimposed dot $\dot{\left(•\right)}$ denotes the time derivative. The shear force field is defined by $T:=-M^{\left(1\right)}:\left[0,L\right]\mapsto R$.

According to the mixture stress-driven model of elasticity [21], the elastic curvature $\chi^{el}$ is a convex combination of the source local field $\;\frac{M}{K}\;$ and of the convolution between the source field and a proper averaging kernel $\phi_{L_{c}}$ described by a characteristic length $L_{c}$. That is,

(3) $$\chi^{el}=\alpha \;\frac{M}{K}\left(x\right)+\left(1-\alpha \right)\;\int_{0}^{L}\phi_{L_{c}}\left(x,\xi \right)\;\frac{M}{K}\left(\xi \right)\;d\xi \;.$$

Denoting with λ a positive nonlocal parameter, the characteristic length is defined as $L_{c}:=\lambda L$. In Equation (3), 0≤α≤1 is the mixture parameter; for α=0, the purely nonlocal stress-driven integral model is obtained, while for α=1, the classic local law of elasticity is recovered.

The averaging kernel is assumed to be the bi-exponential function (Helmholtz’s kernel)

(4) $$\phi_{L_{c}}\left(x\right)=\frac{1}{2L_{c}}\exp\left(-\frac{\left|x\right|}{L_{c}}\right)\;,$$

fulfilling symmetry, positivity and limit impulsivity [13].

Adopting the special kernel in Equation (4), an equivalent differential formulation [14] of the mixture nonlocal model in Equation (3) can be proven as follows. Indeed, the special kernel in Equation (4) is the Green’s function of the linear differential operator defined as $L_{x}:=1-L_{c}^{2}\;{\left(•\right)}^{\left(2\right)}$. Thus, $L_{x}\phi_{L_{c}}\left(x\right)=\delta \left(x\right)$, where δ denotes the Dirac unit impulse. Now, let us rewrite Equation (3) as follows

(5) $$(\chi^{el}-\alpha \;\frac{M}{K})\left(x\right)=\left(1-\alpha \right)\;\int_{0}^{L}\phi_{L_{c}}\left(x,\xi \right)\;\frac{M}{K}\left(\xi \right)\;d\xi \;,$$

and, applying the differential operator $L_{x}$ to Equation (5), we obtain the following expression:

(6) $$L_{x}(\chi^{el}-\alpha \;\frac{M}{K})\left(x\right)=\left(1-\alpha \right)\int_{0}^{L}L_{x}\phi_{L_{c}}\left(x,\xi \right)\;\frac{M}{K}\left(\xi \right)\;d\xi =\left(1-\alpha \right)\frac{M}{K}\left(x\right)\;.$$

Thus, the differential equation equivalent to the two-phase model in Equation (3) writes as

(7) $$L_{x}(\chi^{el}-\alpha \;\frac{M}{K})\left(x\right)=\left(1-\alpha \right)\;\frac{M}{K}\left(x\right)\;.$$

The special averaging kernel satisfies the homogeneous boundary conditions proven in [14]

(8) $$\left\{\begin{array}{c} B_{0}\phi_{L_{c}}{\;|}_{0}=0\;,\\ B_{L}\phi_{L_{c}}{\;|}_{L}=0\;, \end{array}\right.$$

where $B_{0}:=1-L_{c}\;{\left(•\right)}^{\left(1\right)}$ and $B_{L}:=1+L_{c}\;{\left(•\right)}^{\left(1\right)}$ are differential operators defined at the boundary. Thus, the constitutive boundary conditions associated with Equation (7) are obtained by applying $B_{0}$ and $B_{L}$ to Equation (5) as follows:

(9) $$\left\{\begin{array}{c} B_{0}(\chi^{el}-\alpha \;\frac{M}{K}{)\;|}_{0}=0\;,\\ B_{L}(\chi^{el}-\alpha \;\frac{M}{K}{)\;|}_{L}=0\;. \end{array}\right.$$

The equivalent differential Equation (7) equipped with boundary conditions in Equation (9) can be finally rewritten as

(10) $$\frac{\chi (x)}{L_{c}^{2}}-\;\chi^{\left(2\right)}\left(x\right)=\frac{1}{L_{c}^{2}}\frac{M}{K}\left(x\right)-\alpha \;{\frac{M}{K}}^{\left(2\right)}\left(x\right)\;,$$

(11) $$\left\{\begin{array}{c} \chi^{\left(1\right)}\left(0\right)-\frac{1}{L_{c}}\;\chi \left(0\right)=\alpha \;({\frac{M}{K}}^{\left(1\right)}\left(0\right)-\frac{1}{L_{c}}\frac{M}{K}\left(0\right))\;,\\ \chi^{\left(1\right)}\left(L\right)+\frac{1}{L_{c}}\;\chi \left(L\right)=\alpha \;({\frac{M}{K}}^{\left(1\right)}\left(L\right)+\frac{1}{L_{c}}\frac{M}{K}\left(L\right))\;, \end{array}\right.$$

where the geometric curvature field χ has been assumed to be coincident with the elastic one $\chi^{el}$.

## 3. Scale-Dependent Free Vibrations

The structural problem of a Bernoulli–Euler nonlocal beam undergoing free vibrations is formulated by adopting the two-phase stress-driven model illustrated in Section 2.

Let us preliminarily take the second derivative of Equation (10) along the beam abscissa x

(12) $$\;\;\frac{1}{L_{c}^{2}}\chi^{\left(2\right)}-\chi^{\left(4\right)}=\frac{1}{L_{c}^{2}}\;\frac{M^{\left(2\right)}}{K}-\alpha \frac{M^{\left(4\right)}}{K}\;,$$

where a uniform bending stiffness K has been assumed. Enforcing the differential condition of kinematic compatibility in Equation (1) and prescribing the differential equilibrium Equation (2) with a vanishing loading, we obtain the differential equation governing bending-free vibrations of a nonlocal beam, that is

(13) $$\;\;\frac{1}{L_{c}^{2}}\;v^{\left(4\right)}-v^{\left(6\right)}=-\frac{1}{L_{c}^{2}}\;\frac{m\ddot{v}}{K}+\alpha \;m\frac{{\ddot{v}}^{\left(2\right)}}{K}\;.$$

We are interested in synchronous motions v(x,t); that is, each abscissa x of the beam axis executes the same motion in time. From a mathematical point of view, such a solution v(x,t) is separable in spatial and time variables and thus can be expressed as

(14) v(x,t)=ψ(x)ϕ(t).

Substituting Equation (14) in Equation (13) yields

(15) $$\frac{1}{L_{c}^{2}}\psi^{\left(4\right)}\left(x\right)\phi \left(t\right)-\psi^{\left(6\right)}\phi \left(t\right)=-\frac{1}{L_{c}^{2}}\;\frac{m\;\psi \left(x\right)\ddot{\phi }\left(t\right)}{K}+\frac{\alpha \;m\;\psi^{\left(2\right)}\left(x\right)\ddot{\phi }\left(t\right)}{K}\;.$$

Thus, from Equation (15), we obtain the following condition:

(16) $$\frac{\ddot{\phi }\left(t\right)}{\phi (t)}=\frac{\frac{1}{L_{c}^{2}}\psi^{\left(4\right)}\left(x\right)-\psi^{\left(6\right)}\left(x\right)}{-\frac{1}{L_{c}^{2}}\;\frac{m\;\psi (x)}{K}+\frac{\alpha \;m\;\psi^{\left(2\right)}\left(x\right)}{K}}=\beta \;,$$

with β being a constant value. The condition in Equation (16) provides thus two basic differential equations:

(17) $$\left\{\begin{array}{c} \ddot{\phi }\left(t\right)-\beta \;\phi \left(t\right)=0\;,\\ \psi^{\left(6\right)}\left(x\right)-\frac{1}{L_{c}^{2}}\psi^{\left(4\right)}\left(x\right)-\frac{\alpha \;m\;\omega^{2}\psi^{\left(2\right)}\left(x\right)}{K}+\frac{m\;\omega^{2}\psi \left(x\right)}{L_{c}^{2}\;K}=0\;. \end{array}\right.$$

If β were positive, then, according to the general integral of Equation (17)${}_{1}$, ϕ(t) would be the sum of two exponential functions, one of them diverging along with the time variable and thus incompatible with the linearised Bernoulli–Euler theory. Hence, β must be negative and, according to its dimension, it represents the square of natural frequency; that is $\beta :=-\omega^{2}$. Thus, Equation (17)${}_{1}$ is the differential equation governing harmonic motions and its general integral is given by

(18) ϕ(t)=asin(ωt)+bcos(ωt),

where the pair of unknown constants (a,b) can be evaluated by prescribing suitable initial conditions.

Evaluation of beam natural frequencies consists in solving the differential eigenvalue problem formulated as follows:

1. Solving Equation (17)${}_{2}$ in terms of the vector c of integration constants $c_{i},i\in \left\{1,...,6\right\}$.
2. Enforcing standard and constitutive boundary conditions to get a homogeneous algebraic linear system A(λ,α,s)c=o.
3. Solving the characteristic nonlinear equation
  (19) $$\det A_{\left\{\lambda ,\alpha \right\}}\left(\omega^{2}\right)=0\;,$$
  for any fixed pair of parameters {λ,α}, to detect natural frequencies ω.

## 4. Case-Problems: Numerical Outcomes

The differential eigenvalue problem for nonlocal beams illustrated in Section 3 is here adopted to numerically detect fundamental natural frequencies $\omega_{1}$ as functions of nonlocal and mixture parameters, for some exemplar structural schemes: clamped-free, simply supported, clamped-pinned and doubly clamped beams. Plots and results are provided in terms of the following non-dimensional fundamental natural frequency

(20) $$\omega^{*}:=\omega_{1}\;L^{2}\;\sqrt{\frac{m}{K}}\;.$$

The solution methodology explained in Section 3 requires the prescription of four kinematic and/or static boundary conditions depending on the structural scheme at hand. Thus, the following standard boundary conditions will be prescribed:

(21) $$\left\{\begin{array}{ccccc} v=0\;, & \; & v^{\left(1\right)}=0\; & \; & clamped\;end\;,\;\\ v=0\;, & \; & M=0\; & \; & pinned\;end\;,\;\\ M=0\;, & \; & M^{\left(1\right)}=0\; & \; & free\;end\;. \end{array}\right.$$

**Figure 1:**
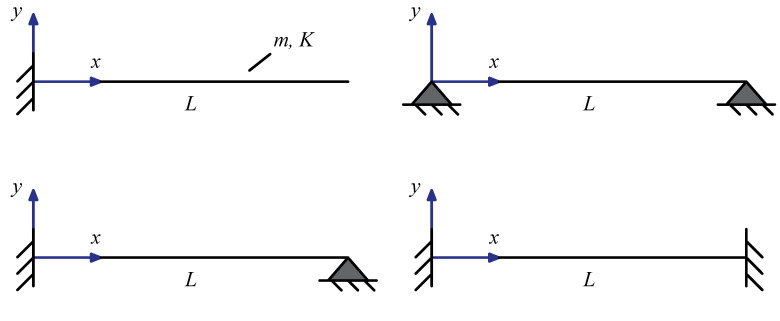
Geometric sketches of adopted structural schemes.

For all considered beams (see Figure 1), the following two constitutive boundary conditions must be enforced:

(22) $$\left\{\begin{array}{cc} \chi^{\left(1\right)}\left(0\right)-\frac{1}{L_{c}}\;\chi \;\left(0\right)\; & =\alpha \;\frac{M^{\left(1\right)}\left(0\right)}{K}-\frac{\alpha }{L_{c}}\frac{M(0)}{K}\;,\\ \chi^{\left(1\right)}\left(L\right)+\frac{1}{L_{c}}\;\chi \;\left(L\right)\; & =\alpha \;\frac{M^{\left(1\right)}\left(L\right)}{K}+\frac{\alpha }{L_{c}}\frac{M(L)}{K}\;. \end{array}\right.$$

It is worth noting that, by virtue of Equation (10), bending moment M appearing in static boundary conditions (Equation (21)) and in constitutive boundary conditions (Equation (22)) can be expressed as follows:

(23) $$M\left(x\right)\;=K\;\psi^{\left(2\right)}\left(x\right)\phi \left(t\right)-L_{c}^{2}\;K\;\psi^{\left(4\right)}\left(x\right)\phi \left(t\right)-\alpha \;L_{c}^{2}\;m\;\psi^{\left(2\right)}\left(x\right)\phi \left(t\right)\;.$$

**Figure 2:**
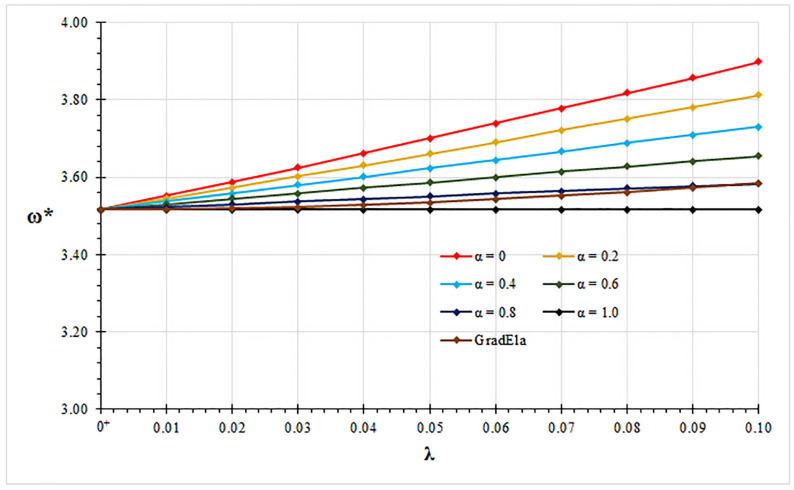
Cantilever beam: non-dimensional fundamental natural frequency $\omega^{*}$ vs. nonlocal parameter λ for α∈{0,0.2,0.4,0.6,0.8,1.0}.

**Figure 3:**
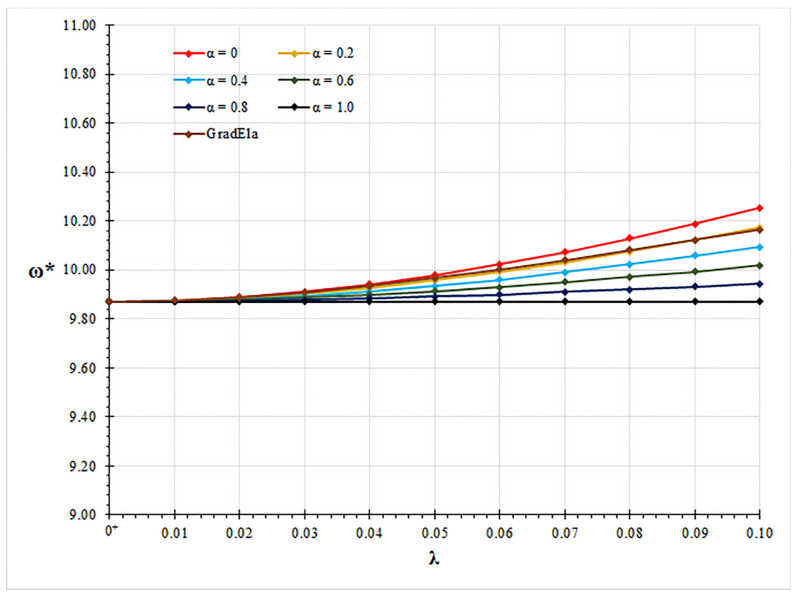
Pinned-pinned beam: non-dimensional fundamental natural frequency $\omega^{*}$ vs. nonlocal parameter λ for α∈{0,0.2,0.4,0.6,0.8,1.0}.

**Figure 4:**
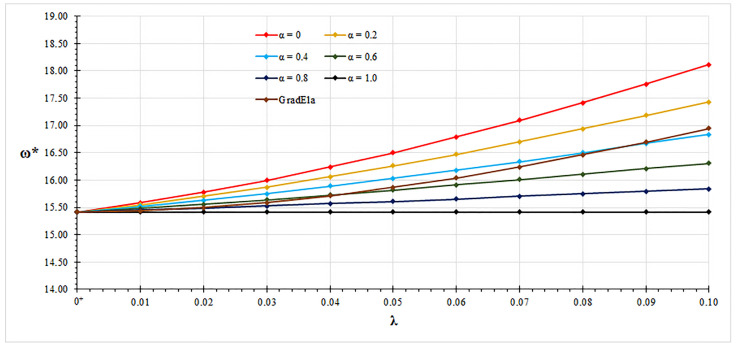
Clamped-pinned beam: non-dimensional fundamental natural frequency $\omega^{*}$ vs. nonlocal parameter λ for α∈{0,0.2,0.4,0.6,0.8,1.0}.

**Figure 5:**
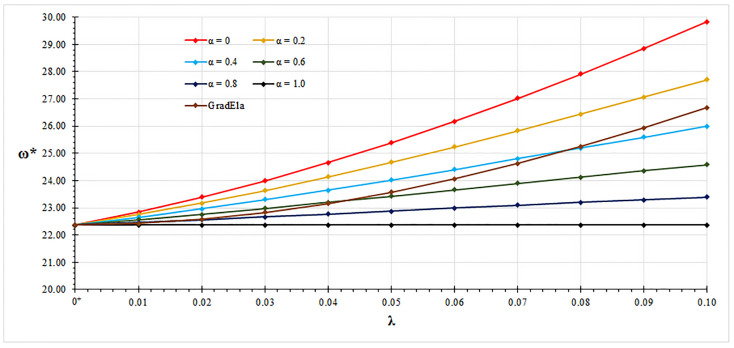
Clamped-clamped beam: non-dimensional fundamental natural frequency $\omega^{*}$ vs. nonlocal parameter λ for α∈{0,0.2,0.4,0.6,0.8,1.0}.

**Figure 6:**
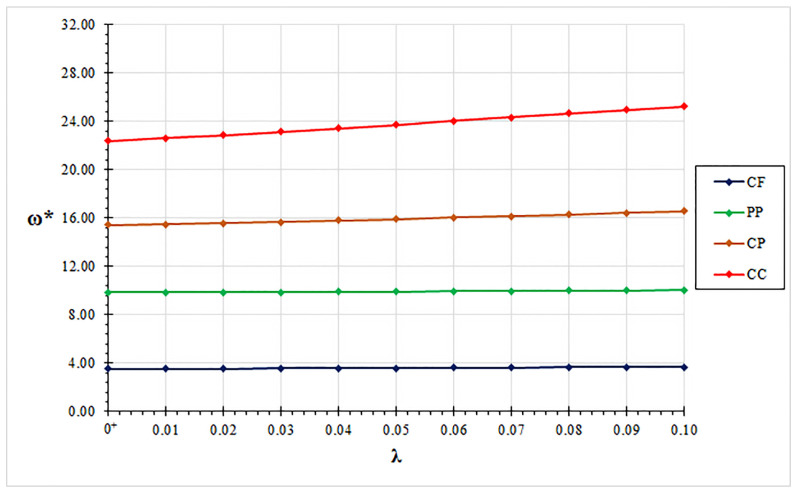
Non-dimensional fundamental natural frequency $\omega^{*}$ vs. nonlocal parameter λ, evaluated for mixture parameter α=0.5, for clamped-free (CP), pinned-pinned (PP), clamped-pinned (CP) and clamped-clamped (CC) structural schemes.

In all examined case studies, free vibration analysis based on the mixture stress-driven elasticity shows stiffening or softening structural behaviors for the increasing nonlocal or mixture parameter, respectively. Indeed, plots of non-dimensional fundamental natural frequencies in Figure 2, Figure 3, Figure 4 and Figure 5 show that frequency increases with λ and decreases with α. Moreover, as shown in Figure 6, stiffening structural behaviours are obtained for increasing redundancy degree. Numerical results of non-dimensional fundamental natural frequencies of adopted structural schemes are shown in Table 1, Table 2, Table 3, Table 4 and Table 5.

It is worth noting that for α=1, local fundamental frequencies are recovered, while for α=0, fundamental natural frequencies obtained by the stress-driven nonlocal model are achieved [28].

Moreover, fundamental natural frequencies are compared with the ones obtained by the gradient elasticity theory [32,33,34]. For all examined case studies, structural responses predicted by gradient elasticity theory (GradEla) are included between purely local (α=1) and nonlocal (α=0) cases, for increasing nonlocal parameter λ.

## 5. Concluding Remarks

Free bending vibration analysis of small-scale elastic beams has been carried out by adopting the stress-driven mixture model of elasticity. In particular, a consistent stress-driven two-phase nonlocal methodology has been presented to parametrically investigate the size-dependent dynamic behaviour of nanobeams. Thus, size effects on fundamental natural frequencies have been numerically investigated and assessed for selected case studies of current interest in nanomechanics, providing new benchmark results for the modelling and design of small-scale structures.

Moreover, the analytical and numerical findings of this research provide an extension of previous contributions in [28], where free vibrations were studied by resorting to the special stress-driven purely nonlocal model [22]. A comparison with the gradient elasticity theory has also been carried out.

Thus, a well-posed methodology has been provided in the paper to capture the size-dependent dynamic behaviours of small-scale structures for a wide range of dynamic nanoengineering problems.

Indeed, the presented local/nonlocal mixture formulation represents a general approach which is able to simulate both softening and stiffening size-dependent dynamic behaviors characterising smaller and smaller technological devices, as experimentally confirmed in the literature (see, e.g., [29,30]). Accordingly, such a strategy can be efficiently exploited for the design and optimisation of nanoengineered materials, modern sensors and actuators.

## Figures and Tables

**Table 1:** Cantilever beam: non-dimensional fundamental natural frequencies $\omega^{*}=\omega_{1}L^{2}\sqrt{\frac{m}{EI}}$.

λ	$\omega^{*}$						GradEla
	α = 0	α = 0.2	α = 0.4	α = 0.6	α = 0.8	α = 1.0	
0${}^{+}$	3.5160	3.5160	3.5160	3.5160	3.5160	3.5160	3.5160
0.01	3.5515	3.5443	3.5372	3.5301	3.5230	3.5160	3.5168
0.02	3.5877	3.5731	3.5585	3.5442	3.5300	3.5160	3.5192
0.03	3.6246	3.6021	3.5800	3.5583	3.5370	3.5160	3.5230
0.04	3.6621	3.6315	3.6016	3.5724	3.5439	3.5160	3.5282
0.05	3.7002	3.6612	3.6232	3.5865	3.5507	3.5160	3.5348
0.06	3.7389	3.6911	3.6449	3.6004	3.5575	3.5160	3.5426
0.07	3.7781	3.7211	3.6666	3.6143	3.5642	3.5160	3.5515
0.08	3.8177	3.7514	3.6882	3.6281	3.5708	3.5160	3.5615
0.09	3.8577	3.7817	3.7098	3.6418	3.5773	3.5160	3.5725
0.10	3.8981	3.8120	3.7313	3.6553	3.5837	3.5160	3.5843

**Table 2:** Pinned-pinned beam: non-dimensional fundamental natural frequencies $\omega^{*}=\omega_{1}L^{2}\sqrt{\frac{m}{EI}}$.

λ	$\omega^{*}$						GradEla
	α = 0	α = 0.2	α = 0.4	α = 0.6	α = 0.8	α = 1.0	
0${}^{+}$	9.8696	9.8696	9.8696	9.8696	9.8696	9.8696	9.8696
0.01	9.8744	9.8734	9.8725	9.8715	9.8706	9.8696	9.8743
0.02	9.8883	9.8845	9.8808	9.8771	9.8733	9.8696	9.8875
0.03	9.9107	9.9025	9.8942	9.8860	9.8778	9.8696	9.9081
0.04	9.9410	9.9266	9.9123	9.8980	9.8838	9.8696	9.9349
0.05	9.9786	9.9565	9.9346	9.9128	9.8911	9.8696	9.9666
0.06	10.0228	9.9916	9.9607	9.9300	9.8997	9.8696	10.0023
0.07	10.0729	10.0313	9.9901	9.9495	9.9093	9.8696	10.0407
0.08	10.1285	10.0751	10.0225	9.9708	9.9198	9.8696	10.0811
0.09	10.1888	10.1225	10.0575	9.9937	9.9311	9.8696	10.1224
0.10	10.2534	10.1731	10.0946	10.0179	9.9429	9.8696	10.1639

**Table 3:** Clamped-pinned beam: non-dimensional fundamental natural frequencies $\omega^{*}=\omega_{1}L^{2}\sqrt{\frac{m}{EI}}$.

λ	$\omega^{*}$						GradEla
	α = 0	α = 0.2	α = 0.4	α = 0.6	α = 0.8	α = 1.0	
0${}^{+}$	15.4184	15.4183	15.4183	15.4183	15.4182	15.4182	15.4182
0.01	15.5854	15.5512	15.5174	15.4840	15.4509	15.4182	15.4385
0.02	15.7781	15.7030	15.6294	15.5575	15.4871	15.4182	15.4969
0.03	15.9958	15.8724	15.7532	15.6379	15.5263	15.4182	15.5897
0.04	16.2373	16.0583	15.8875	15.7242	15.5679	15.4182	15.7137
0.05	16.5016	16.2593	16.0310	15.8154	15.6115	15.4182	15.8657
0.06	16.7874	16.4737	16.1823	15.9105	15.6564	15.4182	16.0427
0.07	17.0931	16.7000	16.3399	16.0085	15.7023	15.4182	16.2419
0.08	17.4173	16.9364	16.5024	16.1084	15.7485	15.4182	16.4606
0.09	17.7585	17.1814	16.6686	16.2092	15.7947	15.4182	16.6965
0.10	18.1152	17.4332	16.8370	16.3103	15.8406	15.4182	16.9473

**Table 4:** Clamped-clamped beam: non-dimensional fundamental natural frequencies $\omega^{*}=\omega_{1}L^{2}\sqrt{\frac{m}{EI}}$.

λ	$\omega^{*}$						GradEla
	α = 0	α = 0.2	α = 0.4	α = 0.6	α = 0.8	α = 1.0	
0${}^{+}$	22.3737	22.3736	22.3736	22.3735	22.3734	22.3733	22.3734
0.01	22.8518	22.7531	22.6559	22.5602	22.4660	22.3733	22.4268
0.02	23.3932	23.1758	22.9655	22.7618	22.5645	22.3733	22.5812
0.03	23.9976	23.6394	23.2993	22.9757	22.6674	22.3733	22.8284
0.04	24.6643	24.1412	23.6540	23.1991	22.7732	22.3733	23.1619
0.05	25.3918	24.6772	24.0259	23.4293	22.8804	22.3733	23.5762
0.06	26.1774	25.2430	24.4106	23.6632	22.9877	22.3733	24.0660
0.07	27.0180	25.8337	24.8038	23.8981	23.0937	22.3733	24.6266
0.08	27.9098	26.4440	25.2014	24.1312	23.1973	22.3733	25.2532
0.09	28.8488	27.0689	25.5994	24.3603	23.2976	22.3733	25.9412
0.10	29.8307	27.7033	25.9944	24.5837	23.3940	22.3733	26.6861

**Table 5:** Non-dimensional fundamental natural frequencies [$\omega^{*}=\omega_{1}L^{2}\sqrt{\frac{m}{EI}}$] of clamped-free (CF), pinned-pinned (PP), clamped-pinned (CP) and clamped-clamped (CC) beams, for α=0.5.

λ	$\omega^{*}$			
	CF	PP	CP	CC
0${}^{+}$	3.5160	9.8696	15.4183	22.3735
0.01	3.5336	9.8720	15.5007	22.6079
0.02	3.5514	9.8789	15.5933	22.8628
0.03	3.5691	9.8901	15.6950	23.1355
0.04	3.5869	9.9051	15.8049	23.4228
0.05	3.6047	9.9237	15.9217	23.7212
0.06	3.6225	9.9453	16.0440	24.0272
0.07	3.6402	9.9697	16.1708	24.3370
0.08	3.6578	9.9965	16.3008	24.6473
0.09	3.6753	10.0254	16.4328	24.9549
0.10	3.6927	10.0560	16.5659	25.2573

## Data Availability

The data presented in this study are available within this article. Further inquiries may be directed to the authors.
